# Whole-Genome Characterization of Epidemic *Neisseria meningitidis* Serogroup C and Resurgence of Serogroup W, Niger, 2015

**DOI:** 10.3201/eid2210.160468

**Published:** 2016-10

**Authors:** Cecilia B. Kretz, Adam C. Retchless, Fati Sidikou, Bassira Issaka, Sani Ousmane, Stephanie Schwartz, Ashley H. Tate, Assimawè Pana, Berthe-Marie Njanpop-Lafourcade, Innocent Nzeyimana, Ricardo Obama Nse, Ala-Eddine Deghmane, Eva Hong, Ola Brønstad Brynildsrud, Ryan T. Novak, Sarah A. Meyer, Odile Ouwe Missi Oukem-Boyer, Olivier Ronveaux, Dominique A. Caugant, Muhamed-Kheir Taha, Xin Wang, the Niger Response Team

**Affiliations:** Centers for Disease Control and Prevention, Atlanta, Georgia, USA (C.B. Kretz, A.C. Retchless, S. Schwartz, A.H. Tate, R.T. Novak, S.A. Meyer, X. Wang);; Centre de Recherche Médicale et Sanitaire, Niamey, Niger (F. Sidikou, B. Issaka, S. Ousmane, O.O.M. Oukem-Boyer);; Agence de Médecine Préventive, Paris (B. Njanpop-Lafourcade);; World Health Organization, Niamey (I. Nzeyimana, A. Pana, R. Obama Nse);; Institut Pasteur, Paris, France (A.-E. Deghmane, E. Hong, M.-K. Taha);; Norwegian Institute of Public Health, Oslo, Norway (O.B. Brynildsrud, D.A. Caugant);; World Health Organization, Geneva, Switzerland (O. Ronveaux)

**Keywords:** Meningococcal meningitis, *Neisseria meningitidis* serogroup C, whole-genome sequencing, Niger, meningitis belt, bacteria

## Abstract

In 2015, Niger reported the largest epidemic of *Neisseria meningitidis* serogroup C (NmC) meningitis in sub-Saharan Africa. The NmC epidemic coincided with serogroup W (NmW) cases during the epidemic season, resulting in a total of 9,367 meningococcal cases through June 2015. To clarify the phylogenetic association, genetic evolution, and antibiotic determinants of the meningococcal strains in Niger, we sequenced the genomes of 102 isolates from this epidemic, comprising 81 NmC and 21 NmW isolates. The genomes of 82 isolates were completed, and all 102 were included in the analysis. All NmC isolates had sequence type 10217, which caused the outbreaks in Nigeria during 2013–2014 and for which a clonal complex has not yet been defined. The NmC isolates from Niger were substantially different from other NmC isolates collected globally. All NmW isolates belonged to clonal complex 11 and were closely related to the isolates causing recent outbreaks in Africa.

*Neisseria meningitidis* commonly causes meningitis in the African meningitis belt, where periodic meningococcal epidemics have contributed to the highest reported incidence of meningococcal meningitis in the world (*1*). Most meningococcal disease historically has been caused by *N. meningitidis* serogroup A (NmA); however, NmA disease dramatically decreased after the preventative MenAfriVac vaccination campaign was initiated in 2010 (*2*). Serogroup W (NmW) has been the major cause of meningococcal disease in the region since then (*2*).

*N. meningitidis* serogroup C (NmC) disease has rarely been reported in the meningitis belt; it has not been detected in many molecular studies of invasive isolates (*3*,*4*) and is rarely found in carriage studies (*5*,*6*). The last large NmC epidemic in Africa occurred in Burkina Faso (then Upper Volta) in 1979 (*7*). During 2013 and 2014, NmC outbreaks were reported in Nigeria (*8*). The Nigerian outbreaks were caused by a novel NmC strain with a previously undescribed sequence type, 10217 (ST-10217), which does not belong to a defined clonal complex. In 2015, an epidemic of 9,367 meningococcal meningitis cases occurred in Niger, with NmC disease comprising most laboratory-confirmed cases (*9*).

NmW disease has been reported in the meningitis belt since the 1980s (*10*,*11*), and NmW from clonal complex 11 (CC11) has been a major concern in the region since 2001 (*12*). The first large epidemic of disease caused by CC11 NmW occurred during 2002 in Burkina Faso (*13*). Subsequently, NmW disease outbreaks were reported in Niger during 2010 and 2011, both involving CC11 (*14*). These outbreaks were followed by another large epidemic caused by CC11 NmW in Burkina Faso during 2012 (*15*). Whole-genome sequencing (WGS) analysis of diverse NmW isolates from around the world has demonstrated that a clone within CC11, commonly associated with NmC, became globally dispersed after it switched to serogroup W (*16*,*17*). WGS analyses also provided sufficient resolution to assign isolates from the meningitis belt to a long-standing regional population and to a clone that became globally dispersed after an outbreak during the 2000 Hajj pilgrimage (*16*,*17*; A. Retchless, unpub. data).

In addition to distinguishing among closely related strains, WGS provides information about allelic variation in genes that may affect antibiotic susceptibility and the coverage of protein-based vaccines. Two vaccines designed for serogroup B meningococcus have been approved for use in the United States and Europe: Trumenba and Bexsero. Trumenba targets the factor H–binding protein (FHbp), and includes components belonging to FHbp subfamilies A and B (*18*). Bexsero includes 4 different components: an FHbp of variant 1 (subfamily B); a *Neisseria* adhesion A protein (NadA); a neisserial heparin-binding antigen (NhbA); and outer membrane vesicles from a serogroup B strain containing PorA P1.4 (*19*). Recognizing the diversity of these genes among strains can aid in evaluating whether these vaccines may provide protection. Likewise, whole-genome sequences can be rapidly screened for indications of antibiotic resistance when the genetic determinants are well characterized, as with genes *penA*, *gyrA,* and *rpoB*, which are involved in reduced susceptibility to penicillin, ciprofloxacin, and rifampin, respectively. To clarify the meningococcal population in Niger during the 2015 epidemic season, we completed genomic analysis on the 102 NmC and NmW invasive isolates collected during this period.

## Materials and Methods

### Strain Collection

**Figure 1:**
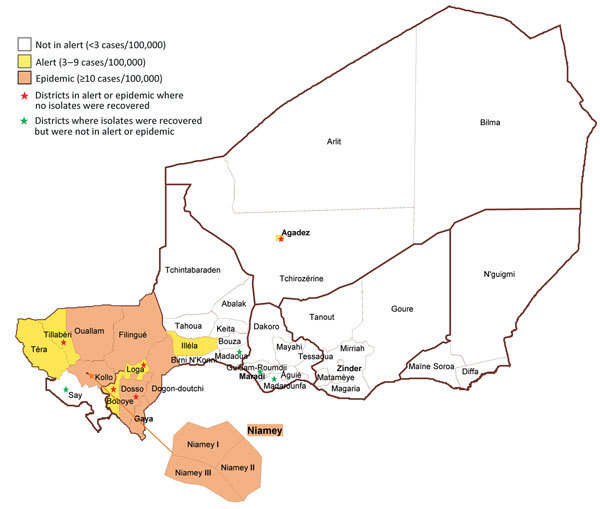
Distribution of *Neisseria meningitidis* isolates by district in Niger during the 2015 epidemic. Dogon-doutchi: 23 (NmC 15; NmW 8); Filingue: 2 (NmC 2); Gaya 2 (NmC 1; NmW 1); Guidan-Roumji: 1 (NmW 1); Illela: 1 (NmC 1); Kollo: 10 (NmC 5; NmW 5); Madaoua: 1 (NmW 1); Madarounfa: 1 (NmW 1); Niamey: 39 (NmC 37; NmW 2); Ouallam: 3 (NmC 2; NmW 1); Say: 3 (NmC 2; NmW 1); Téra: 11 (NmC 11).

A total of 102 isolates from the Centre de Recherche Médicale et Sanitaire (CERMES; Niamey, Niger; Technical Appendix Table 1,) were received at the World Health Organization Collaborating Centres for Meningitis at the following sites: Centers for Disease Control and Prevention (CDC; Atlanta, GA, USA), the Institut Pasteur (Paris, France); and the Norwegian Institute of Public Health (Oslo, Norway). These isolates originated from 12 districts (Figure 1). Species and serogroup of 102 viable isolates were confirmed by culture, PCR, and slide agglutination (*20*) at the 3 World Health Organization Collaborating Centres. The results were 100% in concordance with the laboratory testing performed at CERMES. Conventional PCR-based molecular typing was performed on 9 cerbrospinal fluid specimens for which no associated isolates were available (3 NmW, 6 NmC); all ST, PorA, FetA, and *penA* sequences matched those of isolates that were fully sequenced. An additional 30 NmC isolates from 20 countries and 94 NmW isolates from 15 countries, representing the diversity of the 2 serogroups in the CDC culture collection, were selected and sequenced to compare with the Niger isolates (Technical Appendix Table 1).

### Genome Sequencing

Genome sequencing data for each isolate were generated with both the Pacific Biosystems (PacBio; Meno Park, CA, USA) RSII instrument and the Illumina HiSeq 2500 (San Diego, CA, USA). DNA was extracted from plated isolates by using ArchivePure DNA purification kit (5prime, Gaithersburg, MD, USA). PacBio sequences were generated by using P4-C2 sequencing chemistry and assembled using PacBio’s Hierarchical Genome Assembly Process version 3 (HGAP) (*21*). HGAP produces linear DNA sequences, so we identified circular, complete chromosome sequences on the basis of the existence of reads that bridged the 2 ends of the chromosome after 1 copy of the terminal repeat produced by the assembler was removed. These assemblies were corrected with 250-bp, paired-end Illumina read data generated with TruSeq Rapid SBS chemistry (Illumina) from 600-bp libraries prepared with NEBNext Ultra DNA library preparation kits (New England BioLabs, Ipswich, MA, USA). The Illumina reads were trimmed with Trim Galore version 0.3.7 (Babraham Bioinformatics, Cambridge, UK) to remove reads below Q28, 100 bp, and an error rate of 0.03, then mapped with bowtie version 2.1.0 (*22*) and used to identify base-calling errors and indels by using freebayes version 0.9.16 (https://github.com/ekg/freebayes) with base quality >20, alternate count >20, and coverage >100. The PubMLST (http://pubmlst.org/neisseria/) identifier for the sequences are in Technical Appendix Table 1 and genome coverage information and statistics on each genome in Technical Appendix Table 2.

### Molecular Characterization

We identified multilocus sequence typing (MLST) alleles on the basis of a BLAST (https://blast.ncbi.nlm.nih.gov/Blast.cgi) search of the assembled genomes compared with the PubMLST allele lists (*23*). We also identified potential antibiotic susceptibility based on PubMLST alleles for *gyrA, penA*, and *rpoB* genes. Protein sequences were likewise typed according to PubMLST sequence collection. PorA, PorB, and FetA were classified according to their respective variable regions, NadA was categorized by the Novartis convention of variant and peptide identifier (*24*), NhbA was identified by PubMLST peptide identifier, and FHbp was identified by the PubMLST peptide identifier and the Pfizer peptide identifier (subfamilies A and B).

### Comparative Genomics

For each comparison of genome-wide similarity, we identified single nucleotide polymorphisms (SNPs) using kSNP version 3 software (*25*), with a kmer length of 25. We then built a maximum-likelihood phylogenic tree based on the core SNPs using MEGA6 (*26*), with the Tamura-Nei substitution model and 500 bootstrap iterations.

## Results

### Genomic Characterization and Diversity of NmC Isolates

**Figure 2:**
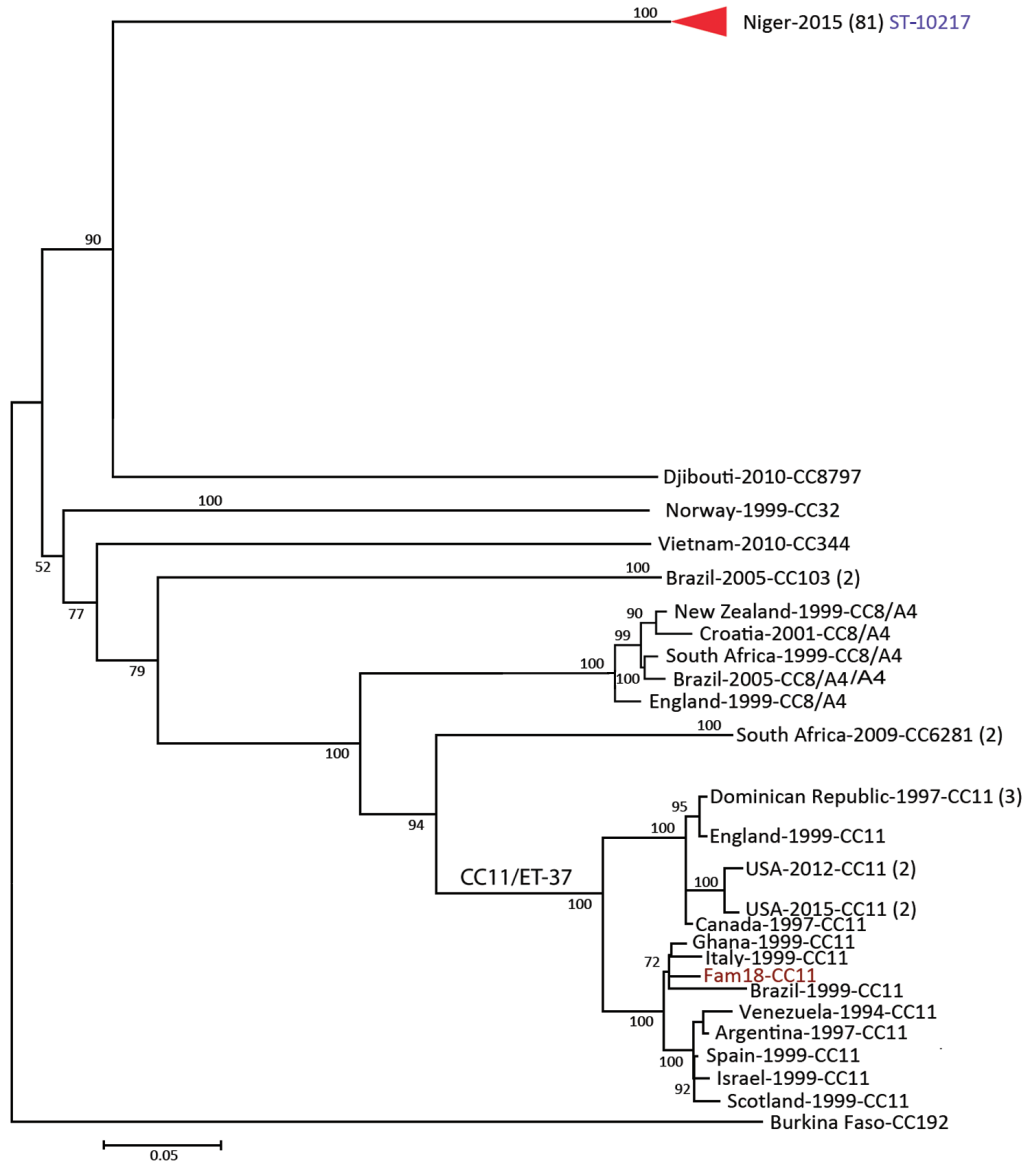
Phylogenetic tree of the *Neisseria meningitidis* serogroup C isolates, labeled with country of origin, year of isolation, and multilocus sequence typing (MLST) group (clonal complex or sequence type). Internal nodes are labeled with bootstrap values. The scale bar is based on the 13,746 positions in the core single nucleotide polymorphism (SNP) matrix and indicates nucleotide substitutions per site.

We sequenced the genomes of 81 Niger NmC isolates using PacBio and Illumina sequencing. PacBio sequencing allowed reconstruction of the complete circular chromosome for 68 isolates. All 81 isolates had the same molecular profile (PorA P1.21–15,16, PorB 3–463, FetA F1–7, and ST-10127, which is not assigned to a known clonal complex; Table). The genome of the ST-10217 isolates were compared with the genomes of NmC isolates from 8 different clonal complexes, collected from countries in North and South America, Europe, Asia, and Africa as far back as 1976. We identified 13,746 core SNPs, with a difference of 0–32 core SNPs between the ST-10217 isolates and a difference of >4,400 core SNPs between ST-10217 and other NmC isolates. The ST-10217 isolates formed a distinct phylogenetic cluster, relative to the other NmC isolates (Figure 2).

**Table:** Summary of molecular typing and serogroups of *Neisseria meningitidis* isolates, Niger, 2015*

Serogroup	No. isolates	ST/CC	PorA†	PorB†	FetA†	NadA‡	NhbA§	FHbp¶	*gyrA*§	*penA*§	*rpoB*§
C	81	ST-10217/NA	P1.21–15,16	3–463	1–7	Not present	798	27/A106	2	22	1
W	14	ST-11/CC11	P1.5,2	2–2	1–1	2/3.6	96	9/B45	4	1	9
W	6	ST-11/CC11	P1.5,2	2–277	1–84	2/3.6	96	9/B45	4	1	9
W	1	ST-11/CC11	P1.5,2	2–60	1–1	2/3.6	96	841/B#	4	1	9
*ST and CC are derived from multilocus sequence typing. CC, clonal complex; NA, not assigned; ST, sequence type. †PorA, PorB, and FetA are typed according to their respective variable regions. ‡NadA is categorized by Novartis conventions of variant and peptide identifier. §The alleles for *gyrA*, *penA*, and *rpoB* are identified by PubMLST DNA allele identifiers (http://pubmlst.org/). NhbA is identified by PubMLST peptide identifier. ¶FHbp is identified by the PubMLST peptide identifier and the Pfizer peptide identifier (subfamilies A and B). Assignment of Pfizer peptide identifier is pending for peptide 841.											

### Genomic Characterization and Diversity of NmW Isolates

**Figure 3:**
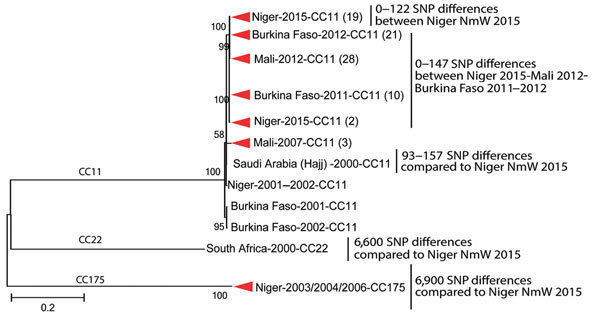
Phylogenetic tree of a subset of the *Neisseria meningitidis* serogroup W (NmW) isolates, labeled with country of origin, year of isolation, and clonal complex (CC). Clades comprising isolates from a single country and year are collapsed, with the isolate count in parentheses. Internal nodes are labeled with bootstrap values, and the number of single nucleotide polymorphisms (SNPs) distinguishing different groups is provided at right. The scale bar is based on the 11,324 positions in the core SNP matrix and indicates nucleotide substitutions per site.

We sequenced the genomes of 21 Niger NmW isolates; 20 were complete circular chromosomes. They all belonged to CC11/ST-11 and had a PorA P1.5,2 (Table). However, they differed in the PorB and FetA sequences. Fourteen NmW isolates had PorB 2–2, six had PorB 2–277, and 1 had PorB 2–60. Fifteen isolates had FetA F1–1, and 6 had FetA F1–84. When we compared the genomes of a collection of African NmW isolates, including CC22 and CC175, we identified 11,324 core SNPs, with a difference of 0–122 SNPs among the isolates from Niger 2015. These isolates were closely related to NmW isolates collected from Burkina Faso and Mali in 2012, with 1–147 SNP differences (Figure 3). Moreover, the Niger CC11 NmW isolates showed 93–157 SNP differences when compared with an isolate collected in Saudi Arabia during the Hajj-related outbreak in 2000.

### Antibiotic-Resistance Genes and Meningococcal Antigen-Encoding Genes

All NmC isolates had *gyr*A allele 2, *pen*A allele 22, and *rpo*B allele 1, whereas all NmW genomes contain *gyr*A 4, *pen*A 1, and *rpo*B 9 (Table). None of these alleles had the mutations associated with resistance to the respective antibiotic (*27*,*28*). The NmC ST-10127 isolates contained FHbp peptide 27, belonging to subfamily A and with 5 aa substitutions relative to peptide 19, against which Trumenba is likely effective on the basis of serum bactericidal activity using human complement (hSBA) (*18*). The NmW isolates contained FHbp peptides 9 or 841 of subfamily B, with 1 aa difference between them and another 13 aa differences relative to peptide 1 (B24), which was susceptible to hSBA (*18*) and is also the FHbp component of the Bexsero vaccine (*19*). None of the NmC isolates contained a *nadA* gene, but the NmW isolates contained NadA peptide 6, which belongs to variant group 2/3 and has 4 aa differences from the Bexsero vaccine component (peptide 8 belonging to variant group 2/3). The NhbA-encoding gene was found in ST-10127 NmC (peptide 798) and ST-11 NmW (peptide 96). Peptide 798 had 68 aa differences relative to the Bexsero component (peptide 2), in addition to being 69 aa longer. Peptide 96 had 85 aa differences, 13 aa missing, and an additional 8 aa relative to peptide 2. No isolate from this epidemic contained PorA P1.4, one of the components of Bexsero.

## Discussion

This study provides a genomic analysis of 102 invasive NmC and NmW strains collected from Niger during a large epidemic in 2015. The isolates within each serogroup (C and W) were closely related and formed a distinct phylogenetic cluster, with identical ST and little variability in the rest of the genome, suggesting a recent emergence, recent clonal expansion, or both. No mutations involved in reduced antibiotic susceptibility were found, suggesting that these isolates are likely susceptible to penicillin, ciprofloxacin, and rifampin. The NmC isolates were not closely related to the reference NmC strain FAM18 or to any of the NmC isolates that were selected from the United States and 20 countries but had the same ST as the strain that caused the outbreaks in Nigeria during 2013–2014 (*8*). The NmW isolates were closely related to isolates collected in the neighboring countries Burkina Faso (2011 and 2012) and Mali (2012). All of these isolates belonged to a clade defined by an isolate from the Hajj-related outbreak in Saudi Arabia in 2000 (A. Retchless, unpub. data), suggesting that the Niger NmW strains may have recently diverged from prior circulating strains in the region. Recent WGS studies have shown that NmW isolates from CC11 form several clades (with 1 harboring the Hajj-related isolates), suggesting a multifocal emergence of the CC11 NmW strains (*16*,*17*; A. Retchless, unpub. data). Researchers may need to analyze larger numbers of NmW isolates from several countries of the meningitis belt to gain knowledge regarding the recent emergence and spread of these strains.

The scale of the epidemic in Niger (>8,500 cases), along with recent NmC outbreaks and sporadic cases in neighboring countries, highlights the risk for resurgent meningococcal meningitis in the meningitis belt, in the form of a newly emergent lineage (*2*). The novelty of this serogroup C lineage is especially concerning, raising questions about how long it has been present in the meningitis belt and why it has not been associated with prior outbreaks. Examination of the PubMLST database revealed only 2 observations of meningococcus with similar profiles: the same ST was observed in serogroup C strains from Nigeria during 2013–2014, and a similar ST (ST-9367, matching at 6 of 7 MLST loci) was represented by a nongroupable isolate from a carriage study in Burkina Faso during 2011. Although comparison between the ST-10127 NmC isolates from Niger 2015 and Nigeria 2013–2014 would reveal recent evolution of ST-10127 lineage, close comparison between ST-10127 and ST-9367 may illuminate recent evolution of the capsule locus. The origin of ST-10127 is unclear due to the limited number of genetically closely related strains. Additional invasive and carriage meningococcal strains that were collected from Africa and other countries in the past few decades should be examined at the genomic level to identify closely related strains and assess the genetic variations that have led to the emerging ST-10127 NmC.

This resurgence of meningococcal disease is not solely due to the novel NmC lineage; laboratory-confirmed NmW cases in Niger increased from 10 in 2013 (*29*) and 14 in 2014 (*2*) to 206 in 2015. The recurrence of non-NmA meningococcal disease after mass vaccination against NmA disease raises questions regarding whether serogroup replacement has occurred and is somehow related to vaccination against NmA, similar to the serotype replacement that was observed after the implementation of pneumococcal vaccines (*30*). Although NmW meningococcal disease continued to resurge after the MenAfriVac campaign in meningitis belt countries began in 2010 (*29*), and NmC subsequently emerged in Nigeria in 2013, neither of these occurrences were likely to be a side-effect of mass vaccination because NmW epidemics had occurred before MenAfriVac (*14*,*31*), and the NmC outbreaks occurred in districts that had not yet been vaccinated. Reemergence of NmC epidemics may have been fueled by the population being immunologically naive to the causative strain. Evaluation of the serogroup replacement hypothesis, and of competing hypotheses such as variation in environmental characteristics (*32*), will greatly benefit from the routine collection of representative molecular surveillance data across the region.

The epidemiologic changes in the meningitis belt underscore the importance of continuous effort to develop vaccines against infectious disease caused by nonvaccine serogroups. Although polysaccharide-based vaccines should provide protection against the NmW and NmC strains, protection may also be provided by recently approved serogroup B meningococcus vaccines that are used in Europe and the United States and target surface proteins that are also found in non-B meningococcal strains (*33*). FHbp-based vaccines can provide protection against strains expressing alleles from the same subfamily of the protein (*34*). The ST-10127 NmC strain contain a FHbp of subfamily A, which is included in the Trumenba bivalent FHbp vaccine, but not the Bexsero multicomponent vaccine (*18*,*19*). This strain lacks NadA but does include a gene encoding NhbA. The ST-11 NmW genomes contain an FHbp of subfamily B, which is included in both Trumenba and Bexsero. Of interest, 3 of the polymorphic residues in FHbp are involved in hydrogen bonding of peptide 1 to human factor H (*35*,*36*). NadA and NhbA are also found in the ST-11 NmW isolates, which suggests that these vaccines may provide protection against ST-11 NmW disease. The antigenicity of these vaccine targets remains to be analyzed to precisely evaluate the coverage of these strains by Trumenba and Bexsero, as has been effectively done for emerging clonal complex 181 serogroup X isolates from the meningitis belt (*37*).

In addition to vaccination, natural immunity could be conferred by prior exposure to *N. meningitidis* strains carrying similar antigens. Because NmC has, until now, been very rare in Africa, immunity against serogroup C is unlikely to exist among the African population, which highlights the urgent need to prepare a response for potential NmC outbreaks and epidemics in the upcoming seasons.

Technical Appendix Details regarding 102 *Neisseria meningitidis* isolates from the Centre de Recherche Médicale et Sanitaire (Niamey, Niger) that were confirmed at World Health Organization Collaborating Centres for Meningitis. An additional 30 NmC isolates from 20 countries and 94 NmW isolates from 15 countries were selected and sequenced to compare with the Niger isolates. Also provided were genome coverage information and statistics for each *Neisseria meningitidis* isolate analyzed, Niger, 2015.
