# Supplementary material for: Whole-Genome Characterization of Epidemic Neisseria meningitidis Serogroup C and Resurgence of Serogroup W, Niger, 2015
Source: Emerg Infect Dis. 2016 Oct;22(10):1762–8. doi: 10.3201/eid2210.160468 (PMC5038424; doi:10.3201/eid2210.160468)
Supplement: Technical Appendix — Details regarding 102 Neisseria meningitidis isolates from the Centre de Recherche Médicale et Sanitaire (Niamey, Niger) that were confirmed at World Health Organization Collaborating Centres for Meningitis. An additional 30 NmC isolates from 20 countries and 94 NmW isolates from 15 countries were selected and sequenced to compare with the Niger isolates. Also provided were genome coverage information and statistics for each Neisseria meningitidis isolate analyzed, Niger, 2015. [file 16-0468-Techapp-s1.pdf]

Article DOI: http://dx.doi.org/10.3201/eid2210.160468

Whole-Genome Characterization of Epidemic *Neisseria meningitidis* Serogroup C and Resurging Serogroup W, Niger, 2015

Technical Appendix

Technical Appendix Table 1. Details regarding 102 *Neisseria meningitidis* isolates from the Centre de Recherche Médicale et sanitaire (Niamey, Niger) that were confirmed at World Health Organization Collaborating Centres for Meninigitis. An additional 30 NmC isolates from 20 countries and 94 NmW isolates from 15 countries, representing the diversity of the 2 serogroups in the CDC culture collection, were selected and sequenced to compare with the Niger isolates.

															gyrA¶		penA¶		rpoB¶		PubMLST
Niger ID	Lab_ID	sAsG	PCR sG	Year	Country	District	ST*	CC*	PorA†	PorB†	FetA†	NadA§	NhbA¶	FHbp#	allele	meaning	allele	meaning	allele	meaning	ID
19-15	M37531	C	NmC	2015	Niger	DOGONDOuTCHI	10217	Unassigned CC for 10217	P1.21-15,16	3-463	1-7	Not present	P00798	A106	2	Sus	22	Sus	1	Sus	39587
82-15	M37609	W	NmW	2015	Niger	KOLLO	11	CC11/ET-37	P1.5,2	2-2	1-1	NadA-2/3.6‡	p0096	B45	4	Sus	1	Sus	9	Sus	39628
83-15	M37610	W	NmW	2015	Niger	KOLLO	11	CC11/ET-37	P1.5,2	2-2	1-1	NadA-2/3.6	p0096	B45	4	Sus	1	Sus	9	Sus	39629
111-15	M37611	W	NmW	2015	Niger	KOLLO	11	CC11/ET-37	P1.5,2	2-2	1-1	NadA-2/3.6	p0096	B45	4	Sus	1	Sus	9	Sus	39630
126-15	M37532	C	NmC	2015	Niger	DOGONDOuTCHI	10217	Unassigned CC for 10217	P1.21-15,16	3-463	1-7	Not present	P00798	A106	2	Sus	22	Sus	1	Sus	39631
171-15	M37612	W	NmW	2015	Niger	OuALLAM	11	CC11/ET-37	P1.5,2	2-2	1-1	NadA-2/3.6	p0096	B45	4	Sus	1	Sus	9	Sus	39632
179-15	M37613	W	NmW	2015	Niger	sAY	11	CC11/ET-37	P1.5,2	2-2	1-1	NadA-2/3.6	p0096	B45	4	Sus	1	Sus	9	Sus	39633
583-15	M37614	W	NmW	2015	Niger	MADAOuA	11	CC11/ET-37	P1.5,2	2-2	1-1	NadA-2/3.6	p0096	B45	4	Sus	1	Sus	9	Sus	39634
587-15	M37533	C	NmC	2015	Niger	NIAMEY	10217	Unassigned CC for 10217	P1.21-15,16	3-463	1-7	Not present	P00798	A106	2	Sus	22	Sus	1	Sus	39635
606-15	M37534	C	NmC	2015	Niger	NIAMEY	10217	Unassigned CC for 10217	P1.21-15,16	3-463	1-7	Not present	P00798	A106	2	Sus	22	Sus	1	Sus	39636
645-15	M37535	C	NmC	2015	Niger	DOGONDOuTCHI	10217	Unassigned CC for 10217	P1.21-15,16	3-463	1-7	Not present	P00798	A106	2	Sus	22	Sus	1	Sus	39637
666-15	M37615	W	NmW	2015	Niger	GuIDAN ROuMJI	11	CC11/ET-37	P1.5,2	2-2	1-1	NadA-2/3.6	p0096	B45	4	Sus	1	Sus	9	Sus	39638
685-15	M37536	C	NmC	2015	Niger	ILLELA	10217	Unassigned CC for 10217	P1.21-15,16	3-463	1-7	Not present	P00798	A106	2	Sus	22	Sus	1	Sus	39639
982-15	NA	C	NmC	2015	Niger	NA	10217	Unassigned CC for 10217	P1.21-15,16	3-463	1-7	Not present	P00798	A106	2	Sus	22	Sus	1	Sus	39640
1010-15	M37616	W	NmW	2015	Niger	DOGONDOuTCHI	11	CC11/ET-37	P1.5,2	2-2	1-1	NadA-2/3.6	p0096	B45	4	Sus	1	Sus	9	Sus	39641
1024-15	M37537	C	NmC	2015	Niger	OuALLAM	10217	Unassigned CC for 10217	P1.21-15,16	3-463	1-7	Not present	P00798	A106	2	Sus	22	Sus	1	Sus	39642
1025-15	M37538	C	NmC	2015	Niger	OuALLAM	10217	Unassigned CC for 10217	P1.21-15,16	3-463	1-7	Not present	P00798	A106	2	Sus	22	Sus	1	Sus	39643
1063-15	M37540	C	NmC	2015	Niger	KOLLO	10217	Unassigned CC for 10217	P1.21-15,16	3-463	1-7	Not present	P00798	A106	2	Sus	22	Sus	1	Sus	39644
1064-15	M37617	W	NmW	2015	Niger	KOLLO	11	CC11/ET-37	P1.5,2	2-277	1-84	NadA-2/3.6	p0096	B45	4	Sus	1	Sus	9	Sus	39645
1077-15	M37541	C	NmC	2015	Niger	DOGONDOuTCHI	10217	Unassigned CC for 10217	P1.21-15,16	3-463	1-7	Not present	P00798	A106	2	Sus	22	Sus	1	Sus	39646
1082-15	M37618	W	NmW	2015	Niger	DOGONDOuTCHI	11	CC11/ET-37	P1.5,2	2-2	1-1	NadA-2/3.6	p0096	B45	4	Sus	1	Sus	9	Sus	39647

Niger ID	Lab_ID	sAsG	PCR sG	Year	Country	District	ST*	CC*	PorA†	PorB†	FetA†	NadA§	NhbA¶	FHbp#	gyrA¶ allele	gyrA¶ meaning	penA¶ allele	penA¶ meaning	rpoB¶ allele	rpoB¶ meaning	PubMLST ID
1078-15	M37542	C	NmC	2015	Niger	DOGONDOuTCHI	10217	Unassigned CC for 10217	P1.21-15,16	3-463	1-7	Not present	P00798	A106	2	Sus	22	Sus	1	Sus	39648
1083-15	M37619	W	NmW	2015	Niger	DOGONDOuTCHI	11	CC11/ET-37	P1.5,2	2-277	1-84	NadA-2/3.6	p0096	B45	4	Sus	1	Sus	9	Sus	39649
1090-15	M37620	W	NmW	2015	Niger	DOGONDOuTCHI	11	CC11/ET-37	P1.5,2	2-277	1-84	NadA-2/3.6	p0096	B45	4	Sus	1	Sus	9	Sus	39650
1096-15	M37621	W	NmW	2015	Niger	DOGONDOuTCHI	11	CC11/ET-37	P1.5,2	2-60	1-1	NadA-2/3.6	p0096	B#	4	Sus	1	Sus	9	Sus	39651
1098-15	M37543	C	NmC	2015	Niger	DOGONDOuTCHI	10217	Unassigned CC for 10217	P1.21-15,16	3-463	1-7	Not present	P00798	A106	2	Sus	22	Sus	1	Sus	39652
1099-15	M37544	C	NmC	2015	Niger	DOGONDOuTCHI	10217	Unassigned CC for 10217	P1.21-15,16	3-463	1-7	Not present	P00798	A106	2	Sus	22	Sus	1	Sus	39653
1103-15	M37545	C	NmC	2015	Niger	DOGONDOuTCHI	10217	Unassigned CC for 10217	P1.21-15,16	3-463	1-7	Not present	P00798	A106	2	Sus	22	Sus	1	Sus	39654
1168-15	NA	C	NmC	2015	Niger	NA	10217	Unassigned CC for 10217	P1.21-15,16	3-463	1-7	Not present	P00798	A106	2	Sus	22	Sus	1	Sus	39655
1169-15	M37546	C	NmC	2015	Niger	TERA	10217	Unassigned	P1.21-15,16	3-463	1-7	Not present	P00798	A106	2	Sus	22	Sus	1	Sus	39656
1172-15	M37547	C	NmC	2015	Niger	TERA	10217	Unassigned CC for 10217	P1.21-15,16	3-463	1-7	Not present	P00798	A106	2	Sus	22	Sus	1	Sus	39657
1173-15	M37548	C	NmC	2015	Niger	KOLLO	10217	Unassigned CC for 10217	P1.21-15,16	3-463	1-7	Not present	P00798	A106	2	Sus	22	Sus	1	Sus	39658
1174-15	M37549	C	NmC	2015	Niger	TERA	10217	Unassigned CC for 10217	P1.21-15,16	3-463	1-7	Not present	P00798	A106	2	Sus	22	Sus	1	Sus	39659
1376-15	M37550	C	NmC	2015	Niger	NIAMEY	10217	Unassigned CC for 10217	P1.21-15,16	3-463	1-7	Not present	P00798	A106	2	Sus	22	Sus	1	Sus	39767
1385-15	M37551	C	NmC	2015	Niger	NIAMEY	10217	Unassigned CC for 10217	P1.21-15,16	3-463	1-7	Not present	P00798	A106	2	Sus	22	Sus	1	Sus	39768
1395-15	NA	C	NmC	2015	Niger	NA	10217	Unassigned CC for 10217	P1.21-15,16	3-463	1-7	Not present	P00798	A106	2	Sus	22	Sus	1	Sus	39769
1396-15	M37552	C	NmC	2015	Niger	NIAMEY	10217	Unassigned CC for 10217	P1.21-15,16	3-463	1-7	Not present	P00798	A106	2	Sus	22	Sus	1	Sus	39770
1434-15	M37553	C	NmC	2015	Niger	NIAMEY	10217	Unassigned CC for 10217	P1.21-15,16	3-463	1-7	Not present	P00798	A106	2	Sus	22	Sus	1	Sus	39771
1437-15	M37554	C	NmC	2015	Niger	NIAMEY	10217	Unassigned CC for 10217	P1.21-15,16	3-463	1-7	Not present	P00798	A106	2	Sus	22	Sus	1	Sus	39772
1640-15	M37555	C	NmC	2015	Niger	KOLLO	10217	Unassigned CC for 10217	P1.21-15,16	3-463	1-7	Not present	P00798	A106	2	Sus	22	Sus	1	Sus	39773
1641-15	M37556	C	NmC	2015	Niger	KOLLO	10217	Unassigned CC for 10217	P1.21-15,16	3-463	1-7	Not present	P00798	A106	2	Sus	22	Sus	1	Sus	39774
1658-15	M37622	W	NmW	2015	Niger	NIAMEY	11	CC11/ET-37	P1.5,2	2-2	1-1	NadA-2/3.6	p0096	B45	4	Sus	1	Sus	9	Sus	39775
1659-15	M37557	C	NmC	2015	Niger	NIAMEY	10217	Unassigned CC for 10217	P1.21-15,16	3-463	1-7	Not present	P00798	A106	2	Sus	22	Sus	1	Sus	39776
1660-15	M37558	C	NmC	2015	Niger	NIAMEY	10217	Unassigned CC for 10217	P1.21-15,16	3-463	1-7	Not present	P00798	A106	2	Sus	22	Sus	1	Sus	39777
1661-15	M37559	C	NmC	2015	Niger	NIAMEY	10217	Unassigned CC for 10217	P1.21-15,16	3-463	1-7	Not present	P00798	A106	2	Sus	22	Sus	1	Sus	39778
1662-15	M37560	C	NmC	2015	Niger	NIAMEY	10217	Unassigned CC for 10217	P1.21-15,16	3-463	1-7	Not present	P00798	A106	2	Sus	22	Sus	1	Sus	39779
1663-15	M37561	C	NmC	2015	Niger	NIAMEY	10217	Unassigned CC for 10217	P1.21-15,16	3-463	1-7	Not present	P00798	A106	2	Sus	22	Sus	1	Sus	39780
1847-15	M37623	W	NmW	2015	Niger	NIAMEY	11	CC11/ET-37	P1.5,2	2-2	1-1	NadA-2/3.6	p0096	B45	4	Sus	1	Sus	9	Sus	39781

															gyrA¶		penA¶		rpoB¶		PubMLST
Niger ID	Lab_ID	sAsG	PCR sG	Year	Country	District	ST*	CC*	PorA†	PorB†	FetA†	NadA§	NhbA¶	FHbp#	allele	meaning	allele	meaning	allele	meaning	ID
1898-15	M37562	C	NmC	2015	Niger	KOLLO	10217	Unassigned CC for 10217	P1.21-15,16	3-463	1-7	Not present	P00798	A106	2	Sus	22	Sus	1	Sus	39782
1945-15	M37563	C	NmC	2015	Niger	NIAMEY	10217	Unassigned CC for 10217	P1.21-15,16	3-463	1-7	Not present	P00798	A106	2	Sus	22	Sus	1	Sus	39783
1946-15	M37564	C	NmC	2015	Niger	NIAMEY	10217	Unassigned CC for 10217	P1.21-15,16	3-463	1-7	Not present	P00798	A106	2	Sus	22	Sus	1	Sus	39784
1947-15	M37565	C	NmC	2015	Niger	NIAMEY	10217	Unassigned CC for 10217	P1.21-15,16	3-463	1-7	Not present	P00798	A106	2	Sus	22	Sus	1	Sus	39785
1956-15	M37566	C	NmC	2015	Niger	NIAMEY	10217	Unassigned CC for 10217	P1.21-15,16	3-463	1-7	Not present	P00798	A106	2	Sus	22	Sus	1	Sus	39786
2007-15	M37567	C	NmC	2015	Niger	DOGONDOuTCHI	10217	Unassigned CC for 10217	P1.21-15,16	3-463	1-7	Not present	P00798	A106	2	Sus	22	Sus	1	Sus	39787
2014-15	M37568	C	NmC	2015	Niger	DOGONDOuTCHI	10217	Unassigned CC for 10217	P1.21-15,16	3-463	1-7	Not present	P00798	A106	2	Sus	22	Sus	1	Sus	39788
2015-15	M37569	C	NmC	2015	Niger	DOGONDOuTCHI	10217	Unassigned CC for 10217	P1.21-15,16	3-463	1-7	Not present	P00798	A106	2	Sus	22	Sus	1	Sus	39789
2023-15	M37570	C	NmC	2015	Niger	DOGONDOuTCHI	10217	Unassigned CC for 10217	P1.21-15,16	3-463	1-7	Not present	P00798	A106	2	Sus	22	Sus	1	Sus	39790
2029-15	M37624	W	NmW	2015	Niger	DOGONDOuTCHI	11	CC11/ET-37	P1.5,2	2-277	1-84	NadA-2/3.6	p0096	B45	4	Sus	1	Sus	9	Sus	39791
2043-15	M37571	C	NmC	2015	Niger	DOGONDOuTCHI	10217	Unassigned CC for 10217	P1.21-15,16	3-463	1-7	Not present	P00798	A106	2	Sus	22	Sus	1	Sus	39792
2081-15	M37572	C	NmC	2015	Niger	FILINGuE	10217	Unassigned CC for 10217	P1.21-15,16	3-463	1-7	Not present	P00798	A106	2	Sus	22	Sus	1	Sus	39793
2085-15	M37573	C	NmC	2015	Niger	FILINGuE	10217	Unassigned CC for 10217	P1.21-15,16	3-463	1-7	Not present	P00798	A106	2	Sus	22	Sus	1	Sus	39794
2099-15	M37625	W	NmW	2015	Niger	KOLLO	11	CC11/ET-37	P1.5,2	2-2	1-1	NadA-2/3.6	p0096	B45	4	Sus	1	Sus	9	Sus	39795
2137-15	M37574	C	NmC	2015	Niger	DOGONDOuTCHI	10217	Unassigned CC for 10217	P1.21-15,16	3-463	1-7	Not present	P00798	A106	2	Sus	22	Sus	1	Sus	39796
2143-15	M37626	W	NmW	2015	Niger	DOGONDOuTCHI	11	CC11/ET-37	P1.5,2	2-277	1-84	NadA-2/3.6	p0096	B45	4	Sus	1	Sus	9	Sus	39797
2146-15	M37627	W	NmW	2015	Niger	DOGONDOuTCHI	11	CC11/ET-37	P1.5,2	2-277	1-84	NadA-2/3.6	p0096	B45	4	Sus	1	Sus	9	Sus	39798
2380-15	M37575	C	NmC	2015	Niger	NIAMEY	10217	Unassigned CC for 10217	P1.21-15,16	3-463	1-7	Not present	P00798	A106	2	Sus	22	Sus	1	Sus	39799
2385-15	M37576	C	NmC	2015	Niger	TERA	10217	Unassigned CC for 10217	P1.21-15,16	3-463	1-7	Not present	P00798	A106	2	Sus	22	Sus	1	Sus	39800
2398-15	M37578	C	NmC	2015	Niger	TERA	10217	Unassigned CC for 10217	P1.21-15,16	3-463	1-7	Not present	P00798	A106	2	Sus	22	Sus	1	Sus	39801
2399-15	M37579	C	NmC	2015	Niger	TERA	10217	Unassigned CC for 10217	P1.21-15,16	3-463	1-7	Not present	P00798	A106	2	Sus	22	Sus	1	Sus	39802
2400-15	M37580	C	NmC	2015	Niger	TERA	10217	Unassigned CC for 10217	P1.21-15,16	3-463	1-7	Not present	P00798	A106	2	Sus	22	Sus	1	Sus	39803
2401-15	M37581	C	NmC	2015	Niger	TERA	10217	Unassigned CC for 10217	P1.21-15,16	3-463	1-7	Not present	P00798	A106	2	Sus	22	Sus	1	Sus	39804
2404-15	M37582	C	NmC	2015	Niger	TERA	10217	Unassigned CC for 10217	P1.21-15,16	3-463	1-7	Not present	P00798	A106	2	Sus	22	Sus	1	Sus	39805
2405-15	M37583	C	NmC	2015	Niger	TERA	10217	Unassigned CC for 10217	P1.21-15,16	3-463	1-7	Not present	P00798	A106	2	Sus	22	Sus	1	Sus	39806
2441-15	M37584	C	NmC	2015	Niger	NIAMEY	10217	Unassigned CC for 10217	P1.21-15,16	3-463	1-7	Not present	P00798	A106	2	Sus	22	Sus	1	Sus	39807

															gyrA¶		penA¶		rpoB¶		PubMLST
Niger ID	Lab_ID	sAsG	PCR sG	Year	Country	District	ST*	CC*	PorA†	PorB†	FetA†	NadA§	NhbA¶	FHbp#	allele	meaning	allele	meaning	allele	meaning	ID
2464-15	M37585	C	NmC	2015	Niger	NIAMEY	10217	Unassigned CC for 10217	P1.21-15,16	3-463	1-7	Not present	P00798	A106	2	Sus	22	Sus	1	Sus	39808
2491-15	M37586	C	NmC	2015	Niger	NIAMEY	10217	Unassigned CC for 10217	P1.21-15,16	3-463	1-7	Not present	P00798	A106	2	Sus	22	Sus	1	Sus	39809
2548-15	M37587	C	NmC	2015	Niger	NIAMEY	10217	Unassigned CC for 10217	P1.21-15,16	3-463	1-7	Not present	P00798	A106	2	Sus	22	Sus	1	Sus	39810
2586-15	NA	C	NmC	2015	Niger	KOLLO	10217	Unassigned CC for 10217	P1.21-15,16	3-463	1-7	Not present	P00798	A106	2	Sus	22	Sus	1	Sus	39811
2749-15	M37628	W	NmW	2015	Niger	MADAROuNFA	11	CC11/ET-37	P1.5,2	2-2	1-1	NadA-2/3.6	p0096	B45	4	Sus	1	Sus	9	Sus	39812
3180-15	M37589	C	NmC	2015	Niger	NIAMEY	10217	Unassigned CC for 10217	P1.21-15,16	3-463	1-7	Not present	P00798	A106	2	Sus	22	Sus	1	Sus	39813
3217-15	M37590	C	NmC	2015	Niger	NIAMEY	10217	Unassigned CC for 10217	P1.21-15,16	3-463	1-7	Not present	P00798	A106	2	Sus	22	Sus	1	Sus	39814
3411-15	M37591	C	NmC	2015	Niger	NIAMEY	10217	Unassigned CC for 10217	P1.21-15,16	3-463	1-7	Not present	P00798	A106	2	Sus	22	Sus	1	Sus	39815
3412-15	M37592	C	NmC	2015	Niger	NIAMEY	10217	Unassigned CC for 10217	P1.21-15,16	3-463	1-7	Not present	P00798	A106	2	Sus	22	Sus	1	Sus	39816
3422-15	M37593	C	NmC	2015	Niger	NIAMEY	10217	Unassigned CC for 10217	P1.21-15,16	3-463	1-7	Not present	P00798	A106	2	Sus	22	Sus	1	Sus	39817
3479-15	M37594	C	NmC	2015	Niger	DOGONDOuTCHI	10217	Unassigned CC for 10217	P1.21-15,16	3-463	1-7	Not present	P00798	A106	2	Sus	22	Sus	1	Sus	39818
3641-15	M37595	C	NmC	2015	Niger	NIAMEY	10217	Unassigned CC for 10217	P1.21-15,16	3-463	1-7	Not present	P00798	A106	2	Sus	22	Sus	1	Sus	39819
3642-15	M37596	C	NmC	2015	Niger	NIAMEY	10217	Unassigned CC for 10217	P1.21-15,16	3-463	1-7	Not present	P00798	A106	2	Sus	22	Sus	1	Sus	39820
3643-15	M37597	C	NmC	2015	Niger	NIAMEY	10217	Unassigned CC for 10217	P1.21-15,16	3-463	1-7	Not present	P00798	A106	2	Sus	22	Sus	1	Sus	39821
3644-15	M37598	C	NmC	2015	Niger	NIAMEY	10217	Unassigned CC for 10217	P1.21-15,16	3-463	1-7	Not present	P00798	A106	2	Sus	22	Sus	1	Sus	39822
3645-15	M37599	C	NmC	2015	Niger	NIAMEY	10217	Unassigned CC for 10217	P1.21-15,16	3-463	1-7	Not present	P00798	A106	2	Sus	22	Sus	1	Sus	39823
3769-15	M37600	C	NmC	2015	Niger	sAY	10217	Unassigned CC for 10217	P1.21-15,16	3-463	1-7	Not present	P00798	A106	2	Sus	22	Sus	1	Sus	39824
3772-15	M37601	C	NmC	2015	Niger	sAY	10217	Unassigned CC for 10217	P1.21-15,16	3-463	1-7	Not present	P00798	A106	2	Sus	22	Sus	1	Sus	39825
3828-15	M37602	C	NmC	2015	Niger	GAYA	10217	Unassigned CC for 10217	P1.21-15,16	3-463	1-7	Not present	P00798	A106	2	Sus	22	Sus	1	Sus	39826
3833-15	M37629	W	NmW	2015	Niger	GAYA	11	CC11/ET-37	P1.5,2	2-2	1-1	NadA-2/3.6	p0096	B45	4	Sus	1	Sus	9	Sus	39827
3882-15	M37603	C	NmC	2015	Niger	NIAMEY	10217	Unassigned CC for 10217	P1.21-15,16	3-463	1-7	Not present	P00798	A106	2	Sus	22	Sus	1	Sus	39828
3884-15	M37604	C	NmC	2015	Niger	NIAMEY	10217	Unassigned CC for 10217	P1.21-15,16	3-463	1-7	Not present	P00798	A106	2	Sus	22	Sus	1	Sus	39829
3949-15	M37605	C	NmC	2015	Niger	NIAMEY	10217	Unassigned CC for 10217	P1.21-15,16	3-463	1-7	Not present	P00798	A106	2	Sus	22	Sus	1	Sus	39830
3962-15	M37606	C	NmC	2015	Niger	NIAMEY	10217	Unassigned CC for 10217	P1.21-15,16	3-463	1-7	Not present	P00798	A106	2	Sus	22	Sus	1	Sus	39831
4019-15	M37607	C	NmC	2015	Niger	NIAMEY	10217	Unassigned CC for 10217	P1.21-15,16	3-463	1-7	Not present	P00798	A106	2	Sus	22	Sus	1	Sus	39832

															gyrA¶		penA¶		rpoB¶		PubMLST
Niger ID	Lab_ID	sAsG	PCR sG	Year	Country	District	ST*	CC*	PorA†	PorB†	FetA†	NadA§	NhbA¶	FHbp#	allele	meaning	allele	meaning	allele	meaning	ID
4036-15	M37608	C	NmC	2015	Niger	NIAMEY	10217	Unassigned CC for 10217	P1.21-15,16	3-463	1-7	Not present	P00798	A106	2	Sus	22	Sus	1	Sus	39833
4064-15	M37539	C	NmC	2015	Niger	NA	10217	Unassigned CC for 10217	P1.21-15,16	3-463	1-7	Not present	P00798	A106	2	Sus	22	Sus	1	Sus	39834
2395-15	M37577	C	NmC	2015	Niger	NA	10217	Unassigned CC for 10217	P1.21-15,16	3-463	1-7	Not present	P00798	A106	2	Sus	22	Sus	1	Sus	39835
NA	M03473	C	NmC	1995	Argentina	NA	11	CC11/ET-37	NA	NA	NA	NA	NA	NA	NA	NA	NA	NA	NA	NA	39836
NA	M05730	C	NmC	1976	Brazil	NA	11	CC11/ET-37	NA	NA	NA	NA	NA	NA	NA	NA	NA	NA	NA	NA	39837
NA	M13970	C	NmC	2005	Brazil	NA	8	CC8/cluster A4	NA	NA	NA	NA	NA	NA	NA	NA	NA	NA	NA	NA	39838
NA	M13973	C	NmC	2005	Brazil	NA	5122	CC103	NA	NA	NA	NA	NA	NA	NA	NA	NA	NA	NA	NA	39839
NA	M14431	C	NmC	2005	Brazil	NA	3779	CC103	NA	NA	NA	NA	NA	NA	NA	NA	NA	NA	NA	NA	39840
NA	M09596	C	NG	NA	Burkina Faso	NA	192	Unassigned CC for 192	NA	NA	NA	NA	NA	NA	NA	NA	NA	NA	NA	NA	39841
NA	M04874	C	NmC	1997	Canada	NA	11	CC11/ET-37	NA	NA	NA	NA	NA	NA	NA	NA	NA	NA	NA	NA	39842
NA	M08566	C	NmC	2001	Croatia	NA	66	CC8/cluster A4	NA	NA	NA	NA	NA	NA	NA	NA	NA	NA	NA	NA	39843
NA	M22236	C	NmC	2010	Djibouti	NA	8797	Unassigned CC for 8797	NA	NA	NA	NA	NA	NA	NA	NA	NA	NA	NA	NA	39844
NA	M03564	C	NmC	1997	Dominican Republic	NA	11	CC11/ET-37	NA	NA	NA	NA	NA	NA	NA	NA	NA	NA	NA	NA	39845
NA	M05451	C	NmC	1998	Dominican Republic	NA	11	CC11/ET-37	NA	NA	NA	NA	NA	NA	NA	NA	NA	NA	NA	NA	39846
NA	M05831	C	NmC	1998	Dominican Republic	NA	11	CC11/ET-37	NA	NA	NA	NA	NA	NA	NA	NA	NA	NA	NA	NA	39847
NA	M05721	C	NmC	1996	England	NA	8	CC8/Cluster A4	NA	NA	NA	NA	NA	NA	NA	NA	NA	NA	NA	NA	39848
NA	M05731	C	NmC	1993	England	NA	11	CC11/ET-37	NA	NA	NA	NA	NA	NA	NA	NA	NA	NA	NA	NA	39849
NA	M05732	C	NmC	1984	Ghana	NA	11	CC11/ET-37	NA	NA	NA	NA	NA	NA	NA	NA	NA	NA	NA	NA	39850
NA	M05733	C	NmC	1988	Israel	NA	11	CC11/ET-37	NA	NA	NA	NA	NA	NA	NA	NA	NA	NA	NA	NA	39851
NA	M05734	C	NmC	1984	Italy	NA	11	CC11/ET-37	NA	NA	NA	NA	NA	NA	NA	NA	NA	NA	NA	NA	39852
NA	M05726	C	NmC	1994	New Zealand	NA	66	CC8/cluster A4	NA	NA	NA	NA	NA	NA	NA	NA	NA	NA	NA	NA	39853
NA	M05749	C	NmC	1987	Norway	NA	32	CC32/ET-5	NA	NA	NA	NA	NA	NA	NA	NA	NA	NA	NA	NA	39854
NA	M05737	C	NmC	1990	Scotland	NA	11	CC11/ET-37	NA	NA	NA	NA	NA	NA	NA	NA	NA	NA	NA	NA	39855
NA	M05729	C	NmC	1990	South Africa	NA	8	CC8/cluster A4	NA	NA	NA	NA	NA	NA	NA	NA	NA	NA	NA	NA	39856
NA	M14217	C	NmC	2005	South Africa	NA	6281	Unassigned CC for 6281	NA	NA	NA	NA	NA	NA	NA	NA	NA	NA	NA	NA	39857
NA	M19586	C	NmC	2009	South Africa	NA	6281	Unassigned CC for 6281	NA	NA	NA	NA	NA	NA	NA	NA	NA	NA	NA	NA	39858
NA	M05738	C	NmC	1985	Spain	NA	11	CC11/ET-37	NA	NA	NA	NA	NA	NA	NA	NA	NA	NA	NA	NA	39859
NA	M28679	C	NmC	2014	United Kingdom	NA	467	CC269	NA	NA	NA	NA	NA	NA	NA	NA	NA	NA	NA	NA	39860
NA	M00601	C	NmC	1994	Venezuela	NA	11	CC11/ET-37	NA	NA	NA	NA	NA	NA	NA	NA	NA	NA	NA	NA	39861
NA	M22440	C	NmC	2010	Vietnam	NA	344	Unassigned CC for 344	NA	NA	NA	NA	NA	NA	NA	NA	NA	NA	NA	NA	39862
NA	M37654	C	NmC	2015	USA	NA	11	CC11/ET-37	NA	NA	NA	NA	NA	NA	NA	NA	NA	NA	NA	NA	NA

Niger ID	Lab_ID	sAsG	PCR sG	Year	Country	District	ST*	CC*	PorA†	PorB†	FetA†	NadA§	NhbA¶	FHbp#	gyrA¶		penA¶		rpoB¶		PubMLST ID
															allele	meaning	allele	meaning	allele	meaning	
NA	M37398	C	NmC	2015	USA	NA	11	CC11/ET-37	NA	NA	NA	NA	NA	NA	NA	NA	NA	NA	NA	NA	NA
NA	M25683	C	NmC	2012	USA	NA	11	CC11/ET-37	NA	NA	NA	NA	NA	NA	NA	NA	NA	NA	NA	NA	NA
NA	M25684	C	NmC	2012	USA	NA	11	CC11/ET-37	NA	NA	NA	NA	NA	NA	NA	NA	NA	NA	NA	NA	NA
NA	M22722	W	NmW	2001	Algeria	NA	11	CC11/ET-37	NA	NA	NA	NA	NA	NA	NA	NA	NA	NA	NA	NA	NA
NA	M22790	W	NmW	2004	Benin	NA	2881	CC175	NA	NA	NA	NA	NA	NA	NA	NA	NA	NA	NA	NA	NA
NA	M22809	W	NmW	2006	Benin	NA	2881	CC175	NA	NA	NA	NA	NA	NA	NA	NA	NA	NA	NA	NA	NA
NA	M22819	W	NmW	2007	Benin	NA	2881	CC175	NA	NA	NA	NA	NA	NA	NA	NA	NA	NA	NA	NA	NA
NA	M07999	W	NmW	2001	Burkina Faso	NA	11	CC11/ET-37	NA	NA	NA	NA	NA	NA	NA	NA	NA	NA	NA	NA	NA
NA	M08000	W	NmW	2001	Burkina Faso	NA	11	CC11/ET-37	NA	NA	NA	NA	NA	NA	NA	NA	NA	NA	NA	NA	NA
NA	M08001	W	NmW	2001	Burkina Faso	NA	11	CC11/ET-37	NA	NA	NA	NA	NA	NA	NA	NA	NA	NA	NA	NA	NA
NA	M09261	W	NmW	2002	Burkina Faso	NA	11	CC11/ET-37	NA	NA	NA	NA	NA	NA	NA	NA	NA	NA	NA	NA	NA
NA	M09293	W	NmW	2002	Burkina Faso	NA	11	CC11/ET-37	NA	NA	NA	NA	NA	NA	NA	NA	NA	NA	NA	NA	NA
NA	M22797	W	NmW	2004	Burkina Faso	NA	11	CC11/ET-37	NA	NA	NA	NA	NA	NA	NA	NA	NA	NA	NA	NA	NA
NA	M22828	W	NmW	2008	Burkina Faso	NA	2881	CC175	NA	NA	NA	NA	NA	NA	NA	NA	NA	NA	NA	NA	NA
NA	M23413	W	NmW	2011	Burkina Faso	NA	11	CC11/ET-37	NA	NA	NA	NA	NA	NA	NA	NA	NA	NA	NA	NA	NA
NA	M23414	W	NmW	2011	Burkina Faso	NA	11	CC11/ET-37	NA	NA	NA	NA	NA	NA	NA	NA	NA	NA	NA	NA	NA
NA	M23415	W	NmW	2011	Burkina Faso	NA	11	CC11/ET-37	NA	NA	NA	NA	NA	NA	NA	NA	NA	NA	NA	NA	NA
NA	M23422	W	NmW	2011	Burkina Faso	NA	11	CC11/ET-37	NA	NA	NA	NA	NA	NA	NA	NA	NA	NA	NA	NA	NA
NA	M23423	W	NmW	2011	Burkina Faso	NA	11	CC11/ET-37	NA	NA	NA	NA	NA	NA	NA	NA	NA	NA	NA	NA	NA
NA	M23426	W	NmW	2011	Burkina Faso	NA	11	CC11/ET-37	NA	NA	NA	NA	NA	NA	NA	NA	NA	NA	NA	NA	NA
NA	M24705	W	NmW	2010	Burkina Faso	NA	8638	CC175	NA	NA	NA	NA	NA	NA	NA	NA	NA	NA	NA	NA	NA
NA	M24730	W	NmW	2011	Burkina Faso	NA	11	CC11/ET-37	NA	NA	NA	NA	NA	NA	NA	NA	NA	NA	NA	NA	NA
NA	M24731	W	NmW	2011	Burkina Faso	NA	11	CC11/ET-37	NA	NA	NA	NA	NA	NA	NA	NA	NA	NA	NA	NA	NA
NA	M24734	W	NmW	2011	Burkina Faso	NA	11	CC11/ET-37	NA	NA	NA	NA	NA	NA	NA	NA	NA	NA	NA	NA	NA
NA	M25430	W	NmW	2012	Burkina Faso	NA	11	CC11/ET-37	NA	NA	NA	NA	NA	NA	NA	NA	NA	NA	NA	NA	NA
NA	M25431	W	NmW	2012	Burkina Faso	NA	2881	CC175	NA	NA	NA	NA	NA	NA	NA	NA	NA	NA	NA	NA	NA
NA	M25432	W	NmW	2012	Burkina Faso	NA	11	CC11/ET-37	NA	NA	NA	NA	NA	NA	NA	NA	NA	NA	NA	NA	NA

Niger ID	Lab_ID	sAsG	PCR sG	Year	Country	District	ST*	CC*	PorA†	PorB†	FetA†	NadA§	NhbA¶	FHbp#	gyrA¶		penA¶		rpoB¶		PubMLST ID
															allele	meaning	allele	meaning	allele	meaning	
NA	M25433	W	NmW	2012	Burkina Faso	NA	11	CC11/ET-37	NA	NA	NA	NA	NA	NA	NA	NA	NA	NA	NA	NA	NA
NA	M25434	W	NmW	2012	Burkina Faso	NA	11	CC11/ET-37	NA	NA	NA	NA	NA	NA	NA	NA	NA	NA	NA	NA	NA
NA	M25435	W	NmW	2012	Burkina Faso	NA	11	CC11/ET-37	NA	NA	NA	NA	NA	NA	NA	NA	NA	NA	NA	NA	NA
NA	M25436	W	NmW	2012	Burkina Faso	NA	11	CC11/ET-37	NA	NA	NA	NA	NA	NA	NA	NA	NA	NA	NA	NA	NA
NA	M25438	W	NmW	2012	Burkina Faso	NA	11	CC11/ET-37	NA	NA	NA	NA	NA	NA	NA	NA	NA	NA	NA	NA	NA
NA	M25440	W	NmW	2012	Burkina Faso	NA	11	CC11/ET-37	NA	NA	NA	NA	NA	NA	NA	NA	NA	NA	NA	NA	NA
NA	M25456	W	NmW	2012	Burkina Faso	NA	11	CC11/ET-37	NA	NA	NA	NA	NA	NA	NA	NA	NA	NA	NA	NA	NA
NA	M25459	W	NmW	2012	Burkina Faso	NA	11	CC11/ET-37	NA	NA	NA	NA	NA	NA	NA	NA	NA	NA	NA	NA	NA
NA	M25462	W	NmW	2012	Burkina Faso	NA	11	CC11/ET-37	NA	NA	NA	NA	NA	NA	NA	NA	NA	NA	NA	NA	NA
NA	M25467	W	NmW	2012	Burkina Faso	NA	11	CC11/ET-37	NA	NA	NA	NA	NA	NA	NA	NA	NA	NA	NA	NA	NA
NA	M25468	W	NmW	2012	Burkina Faso	NA	11	CC11/ET-37	NA	NA	NA	NA	NA	NA	NA	NA	NA	NA	NA	NA	NA
NA	M25469	W	NmW	2012	Burkina Faso	NA	11	CC11/ET-37	NA	NA	NA	NA	NA	NA	NA	NA	NA	NA	NA	NA	NA
NA	M25470	W	NmW	2012	Burkina Faso	NA	11	CC11/ET-37	NA	NA	NA	NA	NA	NA	NA	NA	NA	NA	NA	NA	NA
NA	M25471	W	NmW	2012	Burkina Faso	NA	11	CC11/ET-37	NA	NA	NA	NA	NA	NA	NA	NA	NA	NA	NA	NA	NA
NA	M25472	W	NmW	2012	Burkina Faso	NA	11	CC11/ET-37	NA	NA	NA	NA	NA	NA	NA	NA	NA	NA	NA	NA	NA
NA	M25473	W	NmW	2012	Burkina Faso	NA	11	CC11/ET-37	NA	NA	NA	NA	NA	NA	NA	NA	NA	NA	NA	NA	NA
NA	M25474	W	NmW	2012	Burkina Faso	NA	11	CC11/ET-37	NA	NA	NA	NA	NA	NA	NA	NA	NA	NA	NA	NA	NA
NA	M25476	W	NmW	2012	Burkina Faso	NA	11	CC11/ET-37	NA	NA	NA	NA	NA	NA	NA	NA	NA	NA	NA	NA	NA
NA	M27559	W	NmW	2011	Burkina Faso	NA	11	CC11/ET-37	NA	NA	NA	NA	NA	NA	NA	NA	NA	NA	NA	NA	NA
NA	M22740	W	NmW	2001	Cameroon	NA	11	CC11/ET-37	NA	NA	NA	NA	NA	NA	NA	NA	NA	NA	NA	NA	NA
NA	M22747	W	NmW	2001	Central African Republic	NA	11	CC11/ET-37	NA	NA	NA	NA	NA	NA	NA	NA	NA	NA	NA	NA	NA
NA	M22748	W	NmW	2001	Central African Republic	NA	11	CC11/ET-37	NA	NA	NA	NA	NA	NA	NA	NA	NA	NA	NA	NA	NA
NA	M22803	W	NmW	2005	Chad	NA	11	CC11/ET-37	NA	NA	NA	NA	NA	NA	NA	NA	NA	NA	NA	NA	NA
NA	M22801	W	NmW	2004	Djibouti	NA	11	CC11/ET-37	NA	NA	NA	NA	NA	NA	NA	NA	NA	NA	NA	NA	NA
NA	M07165	W	NmW	1995	Gambia	NA	11	CC11/ET-37	NA	NA	NA	NA	NA	NA	NA	NA	NA	NA	NA	NA	NA

															gyrA¶		penA¶		rpoB¶		PubMLST
Niger ID	Lab_ID	sAsG	PCR sG	Year	Country	District	ST*	CC*	PorA†	PorB†	FetA†	NadA§	NhbA¶	FHbp#	allele	meaning	allele	meaning	allele	meaning	ID
NA	M07161	W	NmW	1994	Mali	NA	11	CC11/ET-37	NA	NA	NA	NA	NA	NA	NA	NA	NA	NA	NA	NA	NA
NA	M07162	W	NmW	1994	Mali	NA	11	CC11/ET-37	NA	NA	NA	NA	NA	NA	NA	NA	NA	NA	NA	NA	NA
NA	M12752	W	NmW	NA	Mali	NA	11	CC11/ET-37	NA	NA	NA	NA	NA	NA	NA	NA	NA	NA	NA	NA	NA
NA	M22160	W	NmW	2007	Mali	NA	11	CC11/ET-37	NA	NA	NA	NA	NA	NA	NA	NA	NA	NA	NA	NA	NA
NA	M22189	W	NmW	2007	Mali	NA	11	CC11/ET-37	NA	NA	NA	NA	NA	NA	NA	NA	NA	NA	NA	NA	NA
NA	M22191	W	NmW	2007	Mali	NA	11	CC11/ET-37	NA	NA	NA	NA	NA	NA	NA	NA	NA	NA	NA	NA	NA
NA	M25070	W	NmW	2012	Mali	NA	11	CC11/ET-37	NA	NA	NA	NA	NA	NA	NA	NA	NA	NA	NA	NA	NA
NA	M25073	W	NmW	2012	Mali	NA	11	CC11/ET-37	NA	NA	NA	NA	NA	NA	NA	NA	NA	NA	NA	NA	NA
NA	M25074	W	NmW	2012	Mali	NA	11	CC11/ET-37	NA	NA	NA	NA	NA	NA	NA	NA	NA	NA	NA	NA	NA
NA	M25075	W	NmW	2012	Mali	NA	11	CC11/ET-37	NA	NA	NA	NA	NA	NA	NA	NA	NA	NA	NA	NA	NA
NA	M25076	W	NmW	2012	Mali	NA	11	CC11/ET-37	NA	NA	NA	NA	NA	NA	NA	NA	NA	NA	NA	NA	NA
NA	M25077	W	NmW	2012	Mali	NA	11	CC11/ET-37	NA	NA	NA	NA	NA	NA	NA	NA	NA	NA	NA	NA	NA
NA	M25078	W	NmW	2012	Mali	NA	11	CC11/ET-37	NA	NA	NA	NA	NA	NA	NA	NA	NA	NA	NA	NA	NA
NA	M25079	W	NmW	2012	Mali	NA	11	CC11/ET-37	NA	NA	NA	NA	NA	NA	NA	NA	NA	NA	NA	NA	NA
NA	M25080	W	NmW	2012	Mali	NA	11	CC11/ET-37	NA	NA	NA	NA	NA	NA	NA	NA	NA	NA	NA	NA	NA
NA	M25081	W	NmW	2012	Mali	NA	11	CC11/ET-37	NA	NA	NA	NA	NA	NA	NA	NA	NA	NA	NA	NA	NA
NA	M25082	W	NmW	2012	Mali	NA	11	CC11/ET-37	NA	NA	NA	NA	NA	NA	NA	NA	NA	NA	NA	NA	NA
NA	M25086	W	NmW	2012	Mali	NA	11	CC11/ET-37	NA	NA	NA	NA	NA	NA	NA	NA	NA	NA	NA	NA	NA
NA	M25087	W	NmW	2012	Mali	NA	11	CC11/ET-37	NA	NA	NA	NA	NA	NA	NA	NA	NA	NA	NA	NA	NA
NA	M25088	W	NmW	2012	Mali	NA	11	CC11/ET-37	NA	NA	NA	NA	NA	NA	NA	NA	NA	NA	NA	NA	NA
NA	M25089	W	NmW	2012	Mali	NA	11	CC11/ET-37	NA	NA	NA	NA	NA	NA	NA	NA	NA	NA	NA	NA	NA
NA	M25093	W	NmW	2012	Mali	NA	11	CC11/ET-37	NA	NA	NA	NA	NA	NA	NA	NA	NA	NA	NA	NA	NA
NA	M25094	W	NmW	2012	Mali	NA	11	CC11/ET-37	NA	NA	NA	NA	NA	NA	NA	NA	NA	NA	NA	NA	NA
NA	M25100	W	NmW	2012	Mali	NA	11	CC11/ET-37	NA	NA	NA	NA	NA	NA	NA	NA	NA	NA	NA	NA	NA
NA	M25101	W	NmW	2012	Mali	NA	11	CC11/ET-37	NA	NA	NA	NA	NA	NA	NA	NA	NA	NA	NA	NA	NA
NA	M25102	W	NmW	2012	Mali	NA	11	CC11/ET-37	NA	NA	NA	NA	NA	NA	NA	NA	NA	NA	NA	NA	NA
NA	M25103	W	NmW	2012	Mali	NA	11	CC11/ET-37	NA	NA	NA	NA	NA	NA	NA	NA	NA	NA	NA	NA	NA
NA	M25104	W	NmW	2012	Mali	NA	11	CC11/ET-37	NA	NA	NA	NA	NA	NA	NA	NA	NA	NA	NA	NA	NA
NA	M25106	W	NmW	2012	Mali	NA	11	CC11/ET-37	NA	NA	NA	NA	NA	NA	NA	NA	NA	NA	NA	NA	NA
NA	M25107	W	NmW	2012	Mali	NA	11	CC11/ET-37	NA	NA	NA	NA	NA	NA	NA	NA	NA	NA	NA	NA	NA
NA	M25108	W	NmW	2012	Mali	NA	11	CC11/ET-37	NA	NA	NA	NA	NA	NA	NA	NA	NA	NA	NA	NA	NA
NA	M25109	W	NmW	2012	Mali	NA	11	CC11/ET-37	NA	NA	NA	NA	NA	NA	NA	NA	NA	NA	NA	NA	NA
NA	M25110	W	NmW	2012	Mali	NA	11	CC11/ET-37	NA	NA	NA	NA	NA	NA	NA	NA	NA	NA	NA	NA	NA
NA	M25111	W	NmW	2012	Mali	NA	11	CC11/ET-37	NA	NA	NA	NA	NA	NA	NA	NA	NA	NA	NA	NA	NA
NA	M22718	W	NmW	2001	Mauritius	Candos	11	CC11/ET-37	NA	NA	NA	NA	NA	NA	NA	NA	NA	NA	NA	NA	NA
NA	M22759	W	NmW	2001	Niger	Niamey	11	CC11/ET-37	NA	NA	NA	NA	NA	NA	NA	NA	NA	NA	NA	NA	NA
NA	M22769	W	NmW	2002	Niger	Niamey	11	CC11/ET-37	NA	NA	NA	NA	NA	NA	NA	NA	NA	NA	NA	NA	NA
NA	M22783	W	NmW	2003	Niger	Niamey	2881	CC175	NA	NA	NA	NA	NA	NA	NA	NA	NA	NA	NA	NA	NA
NA	M22804	W	NmW	2005	Niger	Niamey	2881	CC175	NA	NA	NA	NA	NA	NA	NA	NA	NA	NA	NA	NA	NA
NA	M22811	W	NmW	2006	Niger	Dosso	2881	CC175	NA	NA	NA	NA	NA	NA	NA	NA	NA	NA	NA	NA	NA
NA	M07149	W	NmW	2000	Saudi Arabia	NA	11	CC11/ET-37	NA	NA	NA	NA	NA	NA	NA	NA	NA	NA	NA	NA	NA
NA	M22772	W	NmW	2002	Senegal	NA	11	CC11/ET-37	NA	NA	NA	NA	NA	NA	NA	NA	NA	NA	NA	NA	NA
NA	M07293	W	NmW	2000	South Africa	NA	22	CC22	NA	NA	NA	NA	NA	NA	NA	NA	NA	NA	NA	NA	NA
NA	M25419	W	NmW	2012	South Africa	NA	11	CC11/ET-37	NA	NA	NA	NA	NA	NA	NA	NA	NA	NA	NA	NA	NA
NA	M22822	W	NmW	2007	Togo	NA	2881	CC175	NA	NA	NA	NA	NA	NA	NA	NA	NA	NA	NA	NA	NA

															gyrA¶		penA¶		rpoB¶		PubMLST
Niger ID	Lab_ID	sAsG	PCR sG	Year	Country	District	ST*	CC*	PorA†	PorB†	FetA†	NadA§	NhbA¶	FHbp#	allele	meaning	allele	meaning	allele	meaning	ID

*CC, clonal complex; CDC, Centers for Disease Control and Prevention; ID, identifier; NA, not applicable; n.a., not assigned; NmC, *N. meningitidis* serogroup C; NmW, *N. meningitidis* serogroup W; Sus, susceptible; ST, sequence type. ST and CC are derived from multilocus sequence typing. The CC for sT-10217 is Not assigned (n.a.).
†PorA, PorB, and FetA are typed according to their respective variable regions.
§NadA is categorized by Novartis conventions of variant and peptide ID.
¶The alleles for gyrA, penA, and rpoB are identified by PubMLsT DNA allele IDs. NhbA is identified by PubMLsT peptide ID..
#FHbp is identified by the PubMLsT peptide ID and the Pfizer peptide ID (subfamilies A and B). Assignment of Pfizer peptide ID is pending for peptide 841.

Technical Appendix Table 2. Genome coverage information and statistics for each *Neisseria meningitidis* isolate analyzed, Niger, 2015

Isolate	Synonym	Bases in contigs	Contig counts	PacBio depth of coverage	Illumina depth of coverage	Circular assembly
M37531	19-15	2175296	1	146.7	464.93	Yes
M37532	126-15	2170282	1	100.2	469.54	Yes
M37533	587-15	2174310	1	120.23	720.66	Yes
M37534	606-15	2174329	1	93.1	662.60	Yes
M37535	645-15	2174366	1	165.83	632.19	Yes
M37536	685-15	2174321	1	140.76	507.78	Yes
M37537	1024-15	2174294	1	189.7	531.74	Yes
M37538	1025-15	2169856	1	176.19	485.66	Yes
M37539	4064_15	2172023	1	133.52	402.72	No
M37540	1063-15	2174321	1	144.12	363.56	Yes
M37541	1077-15	2183924	1	134.68	457.09	Yes
M37542	1078-15	2175816	1	171.5	481.52	Yes
M37543	1098-15	2174300	1	151.45	504.09	Yes
M37544	1099-15	2174220	1	151	425.30	Yes
M37545	1103-15	2174310	1	133.56	457.55	Yes
M37546	1169-15	2174333	1	166.45	523.45	Yes
M37547	1172-15	2174332	1	170.96	369.55	Yes
M37548	1173-15	2174303	1	166.44	337.75	Yes
M37549	1174-15	2172497	1	173.99	415.62	Yes
M37550	1376-15	2174309	1	102.32	402.26	Yes
M37551	1385-15	2174223	1	125.71	434.52	Yes
M37552	1396-15	2168525	1	82.93	564.00	Yes
M37553	1434-15	2166229	1	159.33	668.59	Yes
M37554	1437-15	2166233	1	198.32	523.45	Yes
M37555	1640-15	2174308	1	153.76	417.93	Yes
M37556	1641-15	2173021	1	117.79	401.80	No
M37557	1659-15	2190989	1	144.39	496.26	No
M37558	1660-15	2174302	1	147.05	289.37	Yes
M37559	1661-15	2174328	1	112.79	401.34	Yes
M37560	1662-15	2174310	1	97.41	470.46	Yes
M37561	1663-15	2174326	1	88.62	429.45	Yes
M37562	1898-15	2175143	1	80.88	506.86	Yes
M37563	1945-15	2174332	1	89.62	407.79	Yes
M37564	1946-15	2173715	1	140.09	349.73	Yes
M37565	1947-15	2174336	1	67.39	434.52	Yes
M37566	1956-15	2184360	1	87.51	393.97	No
M37567	2007-15	2175750	1	55.45	935.85	Yes
M37568	2014-15	2185943	1	103.56	415.62	No
M37569	2015-15	2174259	1	91.17	385.67	Yes
M37570	2023-15	2176087	1	118.92	448.34	Yes
M37571	2043-15	2173673	1	112.45	421.15	No
M37572	2081-15	2174241	1	80.19	351.58	Yes
M37573	2085-15	2186255	1	90.36	446.50	No
M37574	2137-15	2174196	1	99.14	417.93	Yes
